# Intrapericardial Delivery of APA-Microcapsules as Promising Stem Cell Therapy Carriers in an Experimental Acute Myocardial Infarction Model

**DOI:** 10.3390/pharmaceutics13111824

**Published:** 2021-11-01

**Authors:** Claudia Báez-Díaz, Virginia Blanco-Blázquez, Francisco Miguel Sánchez-Margallo, Esther López, Helena Martín, Albert Espona-Noguera, Javier G. Casado, Jesús Ciriza, José Luis Pedraz, Verónica Crisóstomo

**Affiliations:** 1 CIBERCV, Instituto de Salud Carlos III, 28029 Madrid, Spain; vblanco@ccmijesususon.com (V.B.-B.); msanchez@ccmijesususon.com (F.M.S.-M.); crisosto@ccmijesususon.com (V.C.); 2 Fundación Centro de Cirugía de Mínima Invasión Jesús Usón, 10071 Cáceres, Spain; elopez@ccmijesususon.com (E.L.); hmartin@ccmijesususon.com (H.M.); 3 Centro de Investigaciones y Estudios Avanzados Lucio Lascaray (CIEA), Laboratorio de Desarrollo y Evaluación de Medicamentos, 01006 Vitoria Gasteiz, Spain; albert.espona@upc.edu (A.E.-N.); joseluis.pedraz@ehu.eus (J.L.P.); 4 CIBER bbn, Instituto de Salud Carlos III, 28029 Madrid, Spain; jeciriza@unizar.es; 5 Immunology Unit-Institute of Molecular Pathology Biomarkers, Veterinary Faculty, University of Extremadura, 10003 Cáceres, Spain; jgarcas@unex.es; 6 Tissue Microenvironment (TME) Lab, Aragón Institute of Engineering Research (I3A), University of Zaragoza, 50018 Zaragoza, Spain

**Keywords:** CDCs, AMI, intrapericardial, microcapsules, APA, swine

## Abstract

The administration of cardiosphere-derived cells (CDCs) after acute myocardial infarction (AMI) is very promising. CDC encapsulation in alginate-poly-l-lysine-alginate (APA) could increase cell survival and adherence. The intrapericardial (IP) approach potentially achieves high concentrations of the therapeutic agent in the infarcted area. We aimed to evaluate IP therapy using a saline vehicle as a control (CON), a dose of 30 × 10^6^ CDCs (CDCs) or APA microcapsules containing 30 × 10^6^ CDCs (APA-CDCs) at 72 h in a porcine AMI model. Magnetic resonance imaging (MRI) was used to determine the left ventricular ejection fraction (LVEF), infarct size (IS), and indexed end diastolic and systolic volumes (EDVi; ESVi) pre- and 10 weeks post-injection. Programmed electrical stimulation (PES) was performed to test arrhythmia inducibility before euthanasia. Histopathological analysis was carried out afterwards. The IP infusion was successful in all animals. At 10 weeks, MRI revealed significantly higher LVEF in the APA-CDC group compared with CON. No significant differences were observed among groups in IS, EDVi, ESVi, PES and histopathological analyses. In conclusion, the IP injection of CDCs (microencapsulated or not) was feasible and safe 72 h post-AMI in the porcine model. Moreover, CDCs APA encapsulation could have a beneficial effect on cardiac function, reflected by a higher LVEF at 10 weeks.

## 1. Introduction

Despite a trend in reduced mortality, cardiovascular diseases (CVD) remain the leading cause of mortality in Europe as a whole. Ischaemic heart disease is responsible for 38% of CVD deaths in females and 44% in males [1]. The burden of CVD is not limited to their high mortality, but these diseases also represent a very high economic and social cost, since the associated hospitalizations, surgical interventions and pharmacological prescriptions, are also on the rise [2].

Therapeutic advances in recent decades have reduced acute mortality after myocardial infarction [3], increasing the proportion of patients with extensive infarction who survive this acute phase but subsequently develop chronic heart failure.

Regenerative therapy based on adult stem cells is positioning itself as a therapeutic option, with experimental and clinical studies supporting its use [4,5]. In recent years, cardiosphere-derived cells (CDCs) have been shown to decrease scar mass, increase viable mass and halt adverse remodelling in the clinical and pre-clinical scenarios [6,7,8,9,10].

Although stem cell therapy has become a very promising approach to improving cardiac function, concerns regarding the safety and potential risks, such as the development of cardiac arrhythmias after cell administration, have also emerged [11,12].

The ability of cell therapy to achieve a beneficial effect will depend, among other things, on the ability of cells to survive and to secrete the various paracrine factors that determine cardioprotection, angiogenesis and activation of endogenous cells [13,14]. The approaches that have been tried to increase cell survival and engrafting are varied, from the administration of very high cell doses [15], to the modification of cells [16] and protecting these cells by encapsulation [17].

In previous studies by our group, we verified that the pericardial fluid represents an optimal medium for cell survival [18], allowing the possibility of achieving a high local concentration of the therapeutic agent [19,20].

Myocardial oedema follows a bimodal pattern during the first week after infarction: While the initial inflammatory wave appears immediately upon reperfusion and dissipates at 24 h, the deferred oedematous wave appears on Day 4 and is maximal at around Day 7 after reperfusion [21]. Accordingly, we hypothesized that the administration of a regenerative therapy at 72 h after acute myocardial infarction could avoid direct exposure of the administered cellular product to the deleterious effect of the inflammatory microenvironment, which could enhance the beneficial effect of the therapy, minimizing the development of heart failure secondary to acute myocardial infarction.

Therefore, in this study, we proposed the intrapericardial administration of CDCs, whether encapsulated or not, in a porcine model of sub-acute myocardial infarction in order to evaluate and compare the therapeutic effect of the injected agents. Furthermore, we explored the possible arrhythmogenicity of the administered therapeutics.

## 2. Materials and Methods

### 2.1. Experimental Protocol

**Figure 1:**
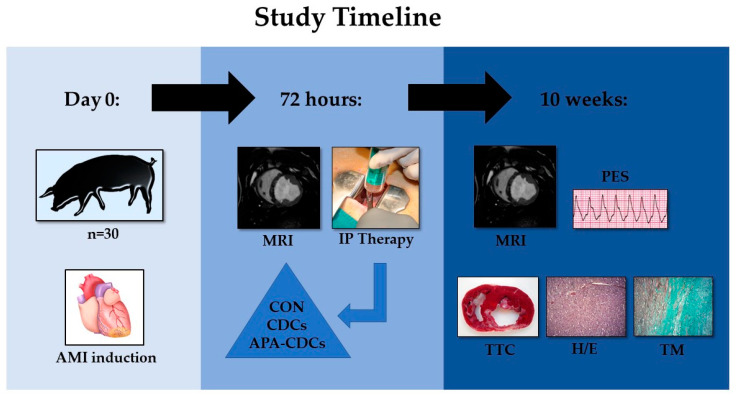
Timeline of the experimental study.

In the present study, 30 young female Large White swine with an initial weight of 35–40 kg were used. All animals underwent a thorough clinical examination and healthy animals were included in the study protocol, which was approved by the Institutional Animal Care and Use Committee (Ref 018/16 and Exp. 20170123-4), while it complied fully with the Directive 2010/63/EU of the European Parliament on the protection of animals used for scientific purposes. The timeline of the experimental study is summarized in Figure 1.

### 2.2. Anaesthesia and Analgesia

Animals were premedicated with diazepam (0.2 mg/kg) and ketamine (15 mg/kg) by the deep intramuscular (IM) route. After 10 min, anaesthesia was induced with 1% propofol (3 mg/kg) intravenously. Subsequently, the animals were intubated with endotracheal tubes of the proper size. Anaesthetic maintenance was carried out using sevoflurane in oxygen (1.8–2% inspiratory fraction). For that purpose, animals were connected to a semi-closed circular circuit attached to a ventilator with an initial fresh gas flow (FGF) of 3 L/min; when the adequate anaesthetic plane was reached, a FGF of 0.5 L/min was established. Ventilation was controlled with a tidal volume of 8–10 mL/kg at an adjusted rate to obtain normocapnia values (35–40 mmHg of CO_2_).

Intraoperative analgesia was achieved by intravenous administration of an association of ketorolac/tramadol (1 mg/kg and 2 mg/kg, respectively) at the beginning of the experience, followed by a continuous infusion of remifentanil (0.15–0.18 μg/kg/h). During anaesthetic maintenance, a continuous infusion of 0.9% NaCl (5–10 mL/kg/h) was administered through a marginal vein of the ear.

Antiarrhythmic therapy was established with 2% lidocaine in a continuous intravenous infusion (1 mg/kg/h), starting after the initial anaesthetic stabilization of the animals and ending after 1 hour of reperfusion after model induction. Prior to coronary occlusion, a bolus of 1 mg/kg of 2% lidocaine was administered.

After infarct creation, the animals were allowed to recover from anaesthesia. As postoperative analgesia, swine received intramuscular buprenorphine at a dose of 10 mg/kg/12 h for 1 day and a 50 μg/h transdermal fentanyl patch was applied.

### 2.3. Infarct Induction

Prior to infarct induction, oral amiodarone (400 mg) was given from 5 days before infarction to 3 days after it. Acetylsalicylic acid (500 mg) was administered from 24 h before model creation until euthanasia. Therapy with clopidogrel was established from 24 h before model induction (300 mg), continuing until euthanasia (75 mg). Furthermore, all pigs received prophylactic antibiotics for 5 days after model induction.

Infarct induction was performed as detailed in a previous study [22]. In brief, anaesthetized pigs were placed in dorsal decubitus in order to establish a percutaneous access to a femoral artery. A coronary angioplasty balloon (Xperience^®^, Ivascular, Barcelona, Spain) of appropriate diameter was placed immediately distal to the origin of the first diagonal branch of the left anterior descending coronary artery and inflated during 90 min. During this occlusion period, possible arrhythmias or ventricular fibrillations were treated by manual chest compressions, 200 J biphasic defibrillation shocks (Zoll M Series Biphasic 200 J, Zoll Medical Corporation, Chelmsford MA, USA) and pharmacological therapy when needed.

Once the balloon had been deflated and removed, haemostasis of the arterial puncture site was performed using manual compression. The animals were kept under general anaesthesia during the reperfusion period to treat possible malignant arrhythmias and were then recovered from anaesthesia and carried to the animal housing facility.

Blood samples were taken for cardiac troponin I (cTnI) analysis and basic blood biochemistry determination before (baseline) and after infarct induction (immediately before IP therapy).

### 2.4. CDCs Isolation and Culture

CDCs were obtained from cardiac tissue explants of the atrial region of a healthy adult male Large White pig as previously described [23]. After washing the tissue samples with a PBS solution, the explants were cut into small fragments (1–2 mm^3^), washed again and subjected to three enzymatic digestions using a 0.2% trypsin solution (Lonza, Basel, Switzerland) and 0.2% Collagenase IV ( Sigma Aldrich, Madrid, Spain in PBS at 37 °C for 5 min each.

Digested tissue fragments were then washed with Complete Explant Medium (CEM) (10% foetal bovine serum (FBS) (Sigma), 1% penicillin-streptomycin (Lonza), 2 mM L-glutamine (Lonza) and 0.2 mM 2-mercaptoethanol (Sigma)) in IMDM (HyClone/Cytiva, Matrid, Spain) with an antifungal and antibiotic supplement and were then cultured in 90 mm Petri plates using the same culture medium at 37 °C and 5% CO_2_.

Three weeks later, tissue fragments were discarded and fibroblast-like cells migrating from the tissue explants were trypsinized and seeded into 30 mm poly-D-lysine coated plates with cardiosphere growing medium (CGM) (10% FBS, 1% penicillin-streptomycin, 2 mM L-glutamine and 0.1 mM 2-mercaptoethanol in 35% IMDM and 65% DMEM-Ham’s F12 (Sigma)). CDCs migrating from the established cardiospheres were selected, seeded again using CGM and expanded (at 37 °C and 5% CO_2_). CDCs at Passages 5 to 10 were used for intrapericardial delivery.

Cells were stored in liquid nitrogen until their usage, using a 90% FBS and 10% dimethyl sulfoxide (DMSO) solution as a freezing medium. For treatment, cells were thawed, centrifuged and resuspended in CGM medium. Subsequently, CDCs were counted, centrifuged again and resuspended in a saline solution at room temperature, adjusting the concentration to 10 × 10^6^ cells/mL. The volume (5 mL) of the cell suspension needed for each injection was pre-filled into syringes that were transported under sterile conditions to the operating room.

### 2.5. Cell Encapsulation

CDCs were incorporated into alginate-poly-l-lysine-alginate (APA) microcapsules using an electrostatic dripper. For that purpose, the cells collected by trypsinization were passed through a 40 µm filter and suspended in a 1.5% sodium alginate solution at a cell density of 6 × 10^6^ cells/mL of alginate. This suspension was deposited in a sterile syringe that was placed in the electrostatic dripper. Using a peristaltic pump, the suspension was passed through a 0.35 mm needle at a flow rate of 5.9 mL/h.

The resulting particles fell into a 55 mM calcium chloride solution, which was kept under stirring for 15 min to ensure that the ionic gelation process was completed. 

Subsequently, the droplets were covered by poly-L-lysine at a concentration of 0.05% for 5 min. After washing them with mannitol, they were covered with a new layer of alginate by incubation with 0.1% alginate for 5 min. The entire process was carried out aseptically and the resulting microcapsules (370 ± 10 µm in diameter and loaded with 4 × 106 cells/mL of alginate) were transferred to the culture medium, where they were kept under standard conditions until use.

### 2.6. Group Allocation and IP Administration

Animals were allocated to control (5 mL saline injection, CON), cell (a dose of 30 × 10^6^ of CDCs suspended in 5 mL, CDCs) or encapsulated cell (a dose of 30 × 10^6^ of CDCs encapsulated in APA and suspended in 5 mL, APA-CDCs) groups before infarct induction. In the three groups, the administration was carried out blindly 72 h after infarction, immediately after acquiring a magnetic resonance imaging (MRI) study.

**Figure 2:**
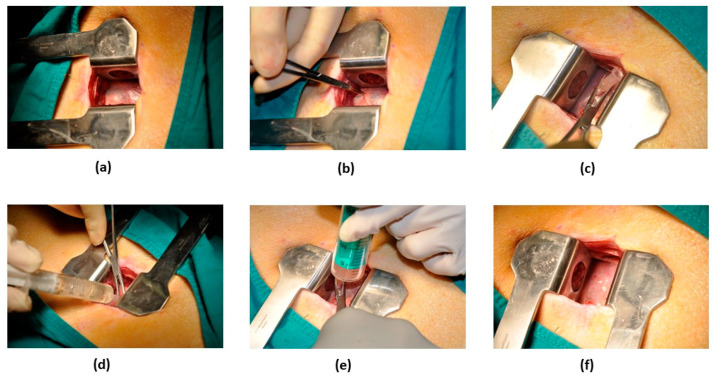
Surgical procedure for IP therapy. (**a**) Exposure of the pericardial sac through a 5-cm-long incision in the fourth or fifth intercostal space. (**b**) Gentle traction of the pericardium to allow puncturing and to gain access to the pericardial cavity. (**c**) Placement of an 18G Abbocath catheter within the pericardium. (**d**) Evacuation of pericardial fluid. (**e**) Slow injection of the therapy through the catheter. (**f**) Appearance of the pericardium after catheter removal.

For IP administration (Figure 2), anesthetized swine were placed in the right lateral decubitus position, allowing access to the thoracic cavity by means of a mini-lateral thoracotomy (≤5 cm). Once the pericardial sac was visible, an 18G Abbocath catheter was inserted inside it and 2–3 mL of pericardial fluid removed before therapy administration. After slow inoculation of the treatment, the catheter was pulled out, the thoracotomy was surgically closed, and the animals were allowed to recover from anaesthesia.

The safety of the IP therapy was evaluated by cTnI analysis after treatment, comparing the results with cTnI values obtained before IP delivery. The occurrence of malignant arrhythmias during IP administration was also explored. Biochemical analysis was carried out at 24 h after IP therapy. During the follow-up period, any signs of infection, bleeding, pain or pericarditis were analysed.

### 2.7. MRI Studies

At 72 h after infarct induction, as well as 10 weeks later, animals were again subjected to general anaesthesia in order to perform MRI examinations as previously described [20]. These studies were carried out with 1.5 T equipment (Philips Intera^®^, Best, The Netherlands) using a specific cardiac 5-element multi-channel coil. The images were obtained with cardiac synchronization based on vectocardiograms and apnoea.

Briefly, breath-hold gradient echo cine mode images in the short axis view were acquired to analyse left ventricular function: Left ventricular ejection fraction (LVEF), end diastolic volume (EDV) and end systolic volume (ESV). EDV and ESV were normalized to body surface area (EDVi and ESVi). Late enhancement images of the myocardial scar using the same cardiac plane were obtained 5–10 min after administration of 0.2 mmol/kg gadobutrol (Gadovist, Bayer, Berlin, Germany) to measure the infarct size (IS). 

### 2.8. End of Study and Post-Mortem Examinations

Ten weeks after infarct creation, immediately after the second MRI study, animals were maintained under deep anaesthesia and subjected to programmed electrical stimulation (PES) by means of a quadrapolar catheter (Marinr SC Steerable Quadrapolar Catheter, Medtronic, Minneapolis, MN, USA) inserted into the left and the right ventricles in order to analyse the inducibility of arrhythmias. Three cycle lengths with up to 4 extrastimuli with different coupling intervals were used following the clinical stimulation protocols. Once the PES was finished, euthanasia was carried out by a lethal dose of potassium chloride (1–2 mmol/kg). Subsequentially, the hearts were explanted and cut into slices 1 cm thick. While one of the sections was incubated at 37 °C for 10 min in a 1% solution of 2,5,3-triphenyl tetrazolium chloride (TTC) in phosphate buffer, samples from the infarct, border and remote areas of the remaining slices were obtained for posterior histopathological analysis by means of haematoxylin-eosin (H/E) and Masson’s trichrome (MT) staining [20].

### 2.9. Statistical Analysis

Animals presenting with LVEF < 45% and IS > 15% (in the pre-treatment MRI examination) were included in the calculations. Data are presented as means ± standard deviations. Normality was checked using the Shapiro-Wilk test. Differences between groups were identified and compared using the Kruskal-Wallis and Mann-Whitney U tests. Intragroup comparisons were calculated with the Wilcoxon paired sample test. Binary data were examined by performing a chi-square test. Values of *p* < 0.05 were considered significant. Calculations were accomplished using the SPSS 18.0 statistical package for Windows (SPSS Inc., Chicago, IL, USA).

## 3. Results

### 3.1. Infarct Induction

One animal died during infarct induction, resulting in the following allocation: CON (*n* = 10), CDCs (*n* = 10) and APA-CDCs (*n* = 9). 

Infarct creation was successfully in all surviving animals, as demonstrated by a significant increase in cTnI values in all groups 72 h after model induction (pre-treatment) (Table 1) (CON: *p* = 0.008; CDCs: *p* = 0.016; APA-CDCs: *p* = 0.008; Wilcoxon paired sample test). On the other hand, no significant differences in cTnI levels were detected among groups either at baseline or after infarction (N.S., Kruskal-Wallis). Basic biochemistry data obtained at baseline and pre-treatment timepoints are presented in Table S1.

### 3.2. IP Therapy Administration

The IP administration was completed successfully in all cases. No malignant arrhythmias occurred during or immediately after IP delivery.

A significant decrease in cTnI levels was seen in the three study groups after therapy administration (CON: *p* = 0.008; CDCs: *p* = 0.008; APA-CDCs: *p* = 0.016; Wilcoxon paired sample test) (Table 1). Nevertheless, no statistically significant differences were observed among groups in this parameter (N.S., Kruskal-Wallis). The results of the biochemical analysis at 24 h post-treatment are presented in Table S1.

During the 10-week follow-up period, no signs of infection, bleeding, pain or pericarditis were detected in any pig.

### 3.3. MRI Studies

The cardiac function parameters are shown in Table 2. One animal belonging to the CDC group exhibited a LVEF of 45% and an IS of 14% in the first MRI study and was therefore excluded from the calculations (CON: *n* = 10; CDCs: *n* = 9, APA-CDCs: *n* = 9). No statistically significant differences were observed among groups before IP administration (N.S., Kruskal-Wallis). 

The post-treatment CMR examination could not be accomplished in two swine: one pig from the CON group that died during MRI acquisition and another one from the APA-CDC group that presented excessive arrhythmias (CON: *n* = 9; CDCs: *n* = 9, APA-CDCs: *n* = 8).

**Figure 3:**
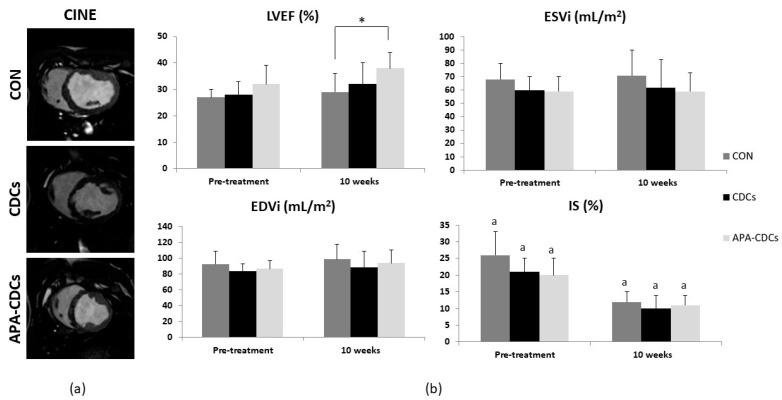
MRI results. (**a**) Representative cine images from the short axis view belonging to each group at 10 weeks. (**b**) Evolution of MRI-derived cardiac function parameters: LVEF, EDVi, ESVi and IS. Differences within groups are denoted by a *p* < 0.05 and those among groups by * *p* < 0.05.

LVEF increased over time in all groups, slightly more so in treated groups, although the differences were not statistically significant (N.S., Kruskal-Wallis). Treatment effects (magnitude of change over time) on LVEF revealed no significant differences among groups (N.S., Kruskal-Wallis) (Figure S1). Nevertheless, at 10 weeks, differences among groups were significant for this parameter (*p* = 0.036, Kruskal-Wallis), owing to the LVEF being significantly higher in the APA-CDC group than in the CON group (*p* = 0.007; Mann-Whitney U test) (Figure 3).

IS decreased significantly over time in the three study groups (CON and CDCs: *p* = 0.004; APA-CDCs: *p* = 0.008; Wilcoxon paired sample test). At the end of the study, however, the differences among groups were not significant (N.S., Kruskal-Wallis). No significant differences were observed in the treatment effects on IS (N.S., Kruskal-Wallis) (Figure S1).

EDVi increased over time in all groups, without reaching statistical significance among them (N.S., Wilcoxon). ESVi increased slightly in the CON and CDC groups (N.S., Wilcoxon), while it remained stable in the APA-CDC group (N.S., Wilcoxon paired sample test). No significant differences among groups were observed regarding ventricular volumes at 10 weeks post-IP administration (N.S., Kruskal-Wallis). Similarly, treatment effects on EDVi and ESVi showed no significant differences among the CON, CDC and APA-CDC groups (N.S., Kruskal-Wallis) (Figure S1). 

### 3.4. End of Study and Post-Mortem Examinations

PES from the left and right ventricles induced ventricular tachycardia in one animal belonging to each group. No significant differences in arrhythmia inducibility among groups (N.S., χ^2^) were seen.

After euthanasia, no pericardial adhesions or treatment-related changes were detected during necropsy and heart explantation.

**Figure 4:**
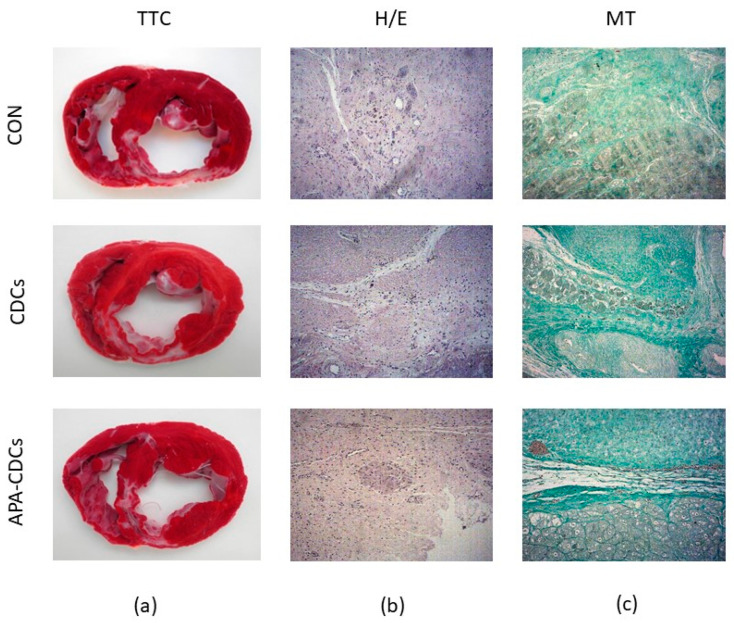
Macroscopical and histopathological appearance of the infarcts. (**a**) TTC staining showing the extension of infarcted tissue in the different groups. (**b**) Representative images from the three study groups of the infarct zone after H/E staining. (**c**) Representative images from the three study groups of the infarct zone after MT staining.

TTC staining revealed transmural fibrous scars with a similar size and site in all animals (Figure 4a). H/E and MT staining of the healthy myocardium showed no alterations, while analysis of the infarct (Figure 4b,c) and border areas revealed lesions such as myocardial necrosis and vascular proliferation, along with extensive myocardial fibrosis in all cases, which presented a lower degree of severity in samples belonging to the border zones. No evident anatomopathological differences among the CON, CDC and APA-CDC groups were therefore observed.

## 4. Discussion

In this blinded, randomized preclinical study, we aimed to evaluate the therapeutic effect of an IP administration of free vs. microencapsulated CDCs. At 10 weeks, we demonstrated an improvement in cardiac function, reflected by a statistically significantly higher LVEF after IP administration of microencapsulated CDCs compared with the control group.

In recent years, heart-derived cells, especially CDCs, have been recognized as an effective cell type for the treatment of ischaemic heart disease [24]. Several preclinical and clinical studies have demonstrated the beneficial effects of these cells in the field of regenerative cardiology [23].

Important limitations of stem cell therapy, however, are poor engraftment and viability rates [25,26]. Early after AMI, a highly adverse inflammatory microenvironment is found, which inhibits the survival of cells transplanted into the myocardium [27]. Although the optimal timepoint of cell delivery has not been determined to date, some previous studies have suggested that optimal cell nesting and survival occurs in the period between Days 3 and 7 post-AMI [28]. Accordingly, in the present study, CDC administration was carried out at 72 h after infarct induction, when an equilibrium between the factors that facilitate and those that hinder cell survival and homing could have been reached [28].

Microencapsulation is a further strategy to increase cell retention and survival, which facilitates allogeneic cell transplantation, since it protects the cells from the host immune reaction [29].

Microcapsules act as a semi-permeable barrier that allows proteins, molecules and other nutrients to diffuse inside the capsules and enable the cells to secrete growth factors and other therapeutic products [25,29,30] that could favour tissue regeneration.

Among the different materials used as building units of microcapsules, APA has been studied the most and is commonly used in microencapsulation. Alginate presents good biocompatibility and low toxicity [31]. The second alginate coating that is used in APA microcapsules reduces immunological reaction after cell implantation [30].

Regarding CDC encapsulation, the recent characterization of these encapsulated cells carried out by our research group demonstrated that APA encapsulation does not alter cell features, keeping long viability (reaching its maximum at Day 21) and growth factor release. The sustained release of growth factors (VEGF, TGF-β1, MSP and IGF-1) from encapsulated CDCs suggests that the therapy with CDC-loaded APA microcapsules could promote angiogenesis and regeneration of the infarcted tissue via the paracrine mechanism [30].

Furthermore, the increased expression of CD117 (related to apoptosis regulation, cell differentiation, proliferation, chemotaxis, cell adhesion and stemness) indicated that encapsulated CDCs could differentiate into more mature cardiac progenitor cells, acquiring the phenotype of cardiac progenitor mast cells, while the decreased expression of Nanog in encapsulated CDCs indicated a lower risk of teratoma formation after transplantation. The higher expression of Hgfl suggested that these encapsulated CDCs are a putative tool for cardiac regeneration, since Hgfl has been related to increased migration, engraftment and commitment in resident cardiac stem cells [30].

In our study, however, histopathological analysis revealed no evident differences among groups neither in the infarct nor in the border areas regarding the presence of necrosis and fibrosis. Similarly, TTC staining revealed infarcts with a similar extent in the three study groups.

Despite the absence of significant differences in macro- and microscopic examinations of the myocardium after euthanasia, a significant enhancement in cardiac function was detected by MRI in animals treated with APA microcapsules containing CDCs at the 10-week timepoint. While LVEF increased over time in the three study groups, this parameter was significantly higher in the APA-CDC group compared with the CON group at the end of the study, suggesting that APA microcapsules represent promising stem cell therapy carriers. A certain degree of progressive recovery of LVEF during the healing process of an AMI (even in untreated subjects) has been identified in earlier studies [32,33] and could most likely be attributed to reversible myocardial dysfunction, known as myocardial stunning [34].

Although LVEF is the most relevant criterion for determining heart function [35,36], other CMR-derived parameters, such as IS and ventricular volumes, have also been proposed as potential surrogate end-points [37].

Regarding IS, in this experimental study, a reduction in the percentage of infarcted myocardium was observed over time, reaching statistical significance in all groups. To our knowledge, the apparent decrease in IS in all study groups, including CON, could be attributed to the dynamic changes that take place in the infarcted myocardium. Early after MI (in our case, before treatment, which was performed 72 h after infarct induction), the presence of oedema can cause an overestimation of IS. This swelling, however, decreases rapidly, resulting in a reduction in the size of infarcted myocardium, even in subjects that were not treated [24,38,39,40,41].

In the present study, the reduction in IS was more remarkable in the CON group, as can be observed in the treatment effect results: −13 ± 6% versus −11 ± 6% in the CDC group versus −9 ± 4% in the APA-CDC group. We believe that this decrease could be explained by a higher degree of LV wall thinning due to adverse ventricular remodelling in the CON group, which causes an augmented loss of cardiomyocytes, destruction of the extracellular matrix of the necrotic area and its replacement by a thin fibrotic scar [42,43], resulting in a lower percentage of infarcted myocardium calculated in the CMR images. In contrast, in the treated groups, the lower percentage of decrease could indicate a protective effect on ventricular remodelling, especially in the APA-CDC group. Nevertheless, further studies, including data on cardiomyocytes, fibrosis or immune cell infiltration or the immune response, will be necessary to validate this point.

Concerning ventricular volumes, increases in EDV and decreases in ESV are common findings after AMI. In this experimental study, EDVi increased in the three groups over time. ESVi increased slightly in the CON and CDC groups over the 10-week period, while it remained stable in the APA-CDC group. According to the literature, these changes in ventricular volume could be attributed to a compensatory hypertrophy of the remote myocardium in order to preserve stroke volume and LVEF [38].

In the present experimental study, we carried out PES in order to identify the risk of developing ventricular arrhythmias due to IP therapy with free or encapsulated CDCs. It has to be taken into account that patients eligible for cell therapy are prone to developing arrhythmias due to their underlying ischaemic heart disease. On the other hand, the potential intrinsic arrhythmogenicity of the delivered stem cells cannot be excluded [44]. In fact, the occurrence of arrhythmias after stem cell therapy is a major concern of the scientific community [44,45]. Different mechanisms, such as immaturity of the electrical phenotypes of the transplanted phenotypes, poor cell–cell coupling and cardiac nerve sprouting, have been proposed as contributors to arrhythmogenic risk after stem cell administration [45]. Despite the proarrhythmic effects that have been attributed to stem cell therapy [46], antiarrhythmic properties have also been reported after mesenchymal stem cell or CDC delivery [47]. In our investigation, however, no statistically significant differences in VT inducibility rates were detected between CON and CDCs-treated animals (both free and encapsulated). Hence, neither pro- nor antiarrhythmic effects related to the therapy could be confirmed in this experimental setting.

Apart from the cell type used, the route of administration seems to further account for the varied results [46]. Certain cell delivery methods have been reported to carry a higher rate of arrhythmias [48]. Thus, intramyocardial injection has been reported to cause enough inflammatory tissue damage and to further increase ventricular irritability [44]. The intrapericardial route, however, seems to provide an alternative approach with less potential for arrhythmias [49].

With regard to IP delivery, additional advantages over other delivery routes, such as homogeneous cell distribution, increased local therapy concentration and limited systemic exposure, have been reported [19,20]. Previous studies by our group demonstrated that the IP approach was safe and feasible for MSCs, CDCs and EVs from CDCs in a chronic and an acute porcine myocardial infarction model [18,23,50]. Additionally, we demonstrated that the intrapericardial administration of EVs from CDCs triggered a M2 polarization during the acute phase of myocardial infarction [49]. Finally, as a further safety evaluation, we demonstrated the absence of changes in proinflammatory cytokines (TFN-α, IL-1β and IFN-α) after intrapericardial CDC administration in the same porcine model, indicating the high safety profile of the therapy [ 50]. Although a cytokine analysis of APA-CDCs was not carried out in either the previous or in the present study, a low immunological response after its administration could be assumed, since alginate-poly-L-lysine-alginate has been reported to reduce immunological reactions after cell implantation [30]. Accordingly, as expected, in this study, we injected 30 × 10^6^ CDCs/animal in the absence of adverse events. Similarly, the IP administration of encapsulated CDCs was uneventful in all cases.

The porcine AMI model is the most attractive one for preclinical studies of myocardial regeneration. However, it is not exempt from certain limitations [24]. Although we carried out comparisons among the CON, CDCs and APA-CDCs groups, an additional group of animals comprising empty APA microcapsules would have been useful for clarifying if APA-CDCs could be able to improve functional results.

It would have been of great interest to explore how well CDCs are incorporated into the myocardial tissues after intrapericardial administration. However, this was not defined in this experimental study and therefore has to be mentioned as a further limitation.

In conclusion, the IP injection of CDCs (microencapsulated or not) is feasible and safe at 72 h post-AMI in the porcine model, as demonstrated by the absence of procedure-related complications. Moreover, CDC encapsulation in APA seems to have a beneficial effect on cardiac function, reflected by a statistically significantly higher LVEF at 10 weeks compared with the CON group, which was, however, not accompanied by a favourable outcome in infarct size reduction. Although no significant histopathological differences among groups were observed, the excellent safety profile, together with the improved cardiac function, could be clinically relevant.

## Figures and Tables

**Table 1:** Cardiac TnI values (µg/L) measured during the study.

Groups	Baseline	Pre-Treatment	Post-Treatment
CON	0.02 ± 0.01 ^a^	5.61 ± 2.56 ^a,b^	2.65 ± 1.69 ^b^
CDCs	0.02 ± 0.01 ^a^	4.83 ± 2.26 ^a,b^	3.04 ± 2.06 ^b^
APA-CDCs	0.02 ± 0.02 ^a^	3.49 ± 2.24 ^a,b^	2.11 ± 0.93 ^b^

Data are presented as means ± standard deviation. Intragroup comparisons at baseline and pre-treatment are denoted by ^a^ *p* < 0.05, and pre-treatment and post-treatment comparisons are denoted by ^b^ *p* < 0.05. No statistically significant differences among groups were detected.

**Table 2:** MRI-derived cardiac function parameters measured before (pre) and 10 weeks after IP treatment, as well as treatment effects.

Groups	CON			CDCs			APA-CDCs		
	Pre	10 Weeks	Treatment Effect	Pre	10 Weeks	Treatment Effect	Pre	10 Weeks	Treatment Effect
LVEF (%)	27 ± 3	29 ± 7 *	2 ± 7	28 ± 5	32 ± 8	3 ± 9	32 ± 7	38 ± 6 *	5 ± 5
IS (%)	26 ± 7 ^a^	12 ± 3 ^a^	−13 ± 6	21 ± 4 ^a^	10 ± 4 ^a^	−11 ± 6	20 ± 5 ^a^	11 ± 3 ^a^	−9 ± 4
EDVi (mL/m^2^)	93 ± 16	99 ± 19	6 ± 24	84 ± 9	89 ± 20	5 ± 21	87 ± 10	94 ± 17	8 ± 10
ESVi (mL/m^2^)	68 ± 12	71 ± 19	3 ± 21	60 ± 8	62 ± 21	2 ± 21	59 ± 11	59 ± 14	2 ± 8

Data are presented as means ± standard deviation. LVEF: left ventricular ejection fraction. IS: Percentage of infarct area of the left ventricle. EDVi: Indexed end diastolic volume. ESVi: Indexed end systolic volume. Differences within groups are denoted by ^a^ *p* < 0.05 and those among groups by * *p* < 0.05.

## Data Availability

Datasets analysed or generated during the study are available from the corresponding author on reasonable request.

## Supplementary Materials

The following are available online at https://www.mdpi.com/article/10.3390/pharmaceutics13111824/s1. Table S1: Evolution of biochemical parameters and reference range for swine in our institution. Figure S1: Treatment effect of MRI-derived cardiac function parameters: LVEF, EDVi, ESVi and IS.
