# Supplementary material for: Intrapericardial Delivery of APA-Microcapsules as Promising Stem Cell Therapy Carriers in an Experimental Acute Myocardial Infarction Model
Source: Pharmaceutics. 2021 Nov 1;13(11):1824. doi: 10.3390/pharmaceutics13111824 (PMC8626005; doi:10.3390/pharmaceutics13111824)
Supplement: Supplementary file 1 [file pharmaceutics-13-01824-s001.zip › pharmaceutics-1338423-supplementary.pdf]

pharmaceutics

Supplementary Material

Intrapericardial Delivery of APA-Microcapsules as Promising Stem Cell Therapy Carriers in an Experimental Acute Myocardial Infarction Model

Claudia Báez-Díaz [1,2,*], Virginia Blanco-Blázquez [1,2], Francisco Miguel Sánchez-Margallo [1,2], Esther López [2], Helena Martín [2], Albert Espona-Noguera [3,4], Javier G. Casado [5], Jesús Ciriza [4,6], José Luis Pedraz [3,4] and Verónica Crisóstomo [1,2]

[1] CIBERCV, Instituto de Salud Carlos III, 28029 Madrid, Spain; vblanco@ccmijesususon.com (V.B.-B.); msanchez@ccmijesususon.com (F.M.S.-M.); crisosto@ccmijesususon.com (V.C.)
[2] Fundación Centro de Cirugía de Mínima Invasión Jesús Usón, 10071 Cáceres, Spain; elopez@ccmijesususon.com (E.L.); hmartin@ccmijesususon.com (H.M.)
[3] Centro de Investigaciones y Estudios Avanzados Lucio Lascaray (CIEA), Laboratorio de Desarrollo y Evaluación de Medicamentos, 01006 Vitoria Gasteiz, Spain; albert.espona@upc.edu (A.E.-N.); joseluis.pedraz@ehu.eus (J.L.P.)
[4] CIBER bbn, Instituto de Salud Carlos III, 28029 Madrid, Spain; jeciriza@unizar.es
[5] Immunology Unit-Institute of Molecular Pathology Biomarkers, Veterinary Faculty, University of Extremadura, 10003 Cáceres, Spain; jgarcas@unex.es
[6] Tissue Microenvironment (TME) Lab, Aragón Institute of Engineering Research (I3A), University of Zaragoza, 50018 Zaragoza, Spain
* Correspondence: cbaez@ccmijesususon.com

Citation: Báez-Díaz, C.; Blanco-Blázquez, V.; Sánchez-Margallo, F.M.; López, E.; Martín, H.; Espona-Noguera, A.; Casado, J.G.; Ciriza, J.; Pedraz, J.L. and Crisóstomo, V. Intrapericardial Delivery of APA-Microcapsules as Promising Stem Cell Therapy Carriers in an Experimental Acute Myocardial Infarction Model. *Pharmaceutics* **2021**, *13*, 1815. https://doi.org/pharmaceutics13111815

Academic Editors: Ana Isabel Flores, Juan Luis Paris and Paz de la Torre

Received: 28 July 2021
Accepted: 15 October 2021
Published: 31 October 2021

Publisher's Note: MDPI stays neutral with regard to jurisdictional claims in published maps and institutional affiliations.

Copyright: © 2021 by the authors. Submitted for possible open access publication under the terms and conditions of the Creative Commons Attribution (CC BY) license (http://creativecommons.org/licenses/by/4.0/).

Table S1. Evolution of biochemical parameters and reference range for swine in our institution. Data presented as mean±standard deviation. Intragroup and Intergroup comparisons at each timepoint are denoted by a or b $p < 0.05$ and * or ** $p < 0.05$, respectively. Bilirubin: In CON significant differences between baseline and 24h post-treatment timepoints, as well as pre-and 24h post-treatment timepoints were observed. No significant differences between groups were detected. Values remained within the reference values for swine in all groups. Creatinine: No significant differences were seen either within groups over time or between groups at the different timepoints in this parameter. Values remained within the reference values for swine in all groups. Glucose: No significant differences were seen either within groups over time or between groups at the different timepoints in this parameter. Values remained within the reference values for swine in all groups. GOT: In CON significant differences between baseline and pre-treatment timepoints as well as between pre and 24h post treatment timepoints were observed. In CDCs significant differences between baseline and 24h post-treatment timepoints as well as between pre-and 24h post-treatment timepoints. In APA-CDCs significant differences between baseline and pre-treatment as well as baseline and 24h post-treatment were detected. No significant differences between groups were seen. GOT levels were increased over the upper range reference level at 24h post-treatment timepoint in all groups. GPT: In the three groups significant differences between groups were found between baseline and pre-treatment timepoints as well as between baseline and 24h post-treatment. No differences between groups were seen. GPT levels were increased over the upper range reference level at pre-treatment and 24h post-treatment timepoints in all groups. Proteins: In CON and CDCs significant differences between baseline and pre-treatment timepoints, as well as between pre and 24h post treatment timepoints were observed. Significant differences between CON and APA-CDCs and CDCs and APA-CDCs were observed in this parameter at pre-treatment timepoint. Values remained within the reference values for swine in all groups. Urea: In APA-CDCs significant differences between groups were found between pre and 24h post-treatment. Significant differences were observed between CON and APA-CDCs at baseline timepoint. Values remained within the reference values for swine in CON and CDCs and increased slightly over the upper range reference level at 24h post-treatment timepoint in APA-CDCs.

**Groups**	**Reference Range**	**CON**			**CDCs**			**APA-CDCs**		
		**Baseline**	**Before Treatment**	**24h after Treatment**	**Baseline**	**Before Treatment**	**24h after Treatment**	**Baseline**	**Before Treatment**	**24h after Treatment**
Bilirubin (mg/dL)	0–0.5	0.5 ± 0.4a	0.2 ± 0.1b	0.1 ± 0.1a,b	0.3 ± 0.2	0.2 ± 0.1	0.2 ± 0.1	0.2 ± 0.1	0.2 ± 0.1	0.2 ± 0.1
Creatinine (mg/dL)	0.8–2.3	1.6 ± 0.3	1.5 ± 0.3	1.5 ± 0.3	1.6 ± 0.3	1.5 ± 0.3	1.5 ± 0.2	1.7 ± 0.6	1.8 ± 0.8	2.2 ± 1.7
Glucose (mg/dL)	60–150	110.2 ± 43.9	88.8 ± 21.3	92.3 ± 20.0	90.0 ± 28.7	77.1 ± 16.0	86.6 ± 19.0	90.5 ± 12.7	116.8 ± 95.6	118.9 ± 70.3
GOT (U/L)	15–80	34.3 ± 9.2a	57.0 ± 22.1a,b	92.3 ± 13.5a,b	33.9 ± 6.9a	63.8 ± 40.3b	105.8 ± 46.4a,b	32.3 ± 7.2a,b	82.1 ± 50.4a	123.0 ± 30.4b
GPT (U/L)	22–60	32.7 ± 9.8a,b	75.0 ± 23.1a	82.7 ± 18.8b	36.2 ± 13.7a,b	76.3 ± 21.1a	86.0 ± 21.5b	32.3 ± 3.2a,b	78.0 ± 35.7a	84.9 ± 29.7b
Proteins (g/dL)	5.8–8.3	6.3 ± 0.5a	5.3 ± 0.4*,a,b	6.3 ± 0.5b	6.2 ± 0.5a	5.1 ± 0.9**,a,b	5.9 ± 1.2b	6.3 ± 0.6	7.2 ± 3.8*,**	8.3 ± 4.4
Urea (mg/L)	10-30	27.4 ± 5.1*	22.3 ± 5.4	27.9 ± 3.9	27.1 ± 13.9	22.9 ± 6.4	25.1 ± 6.6	19.4 ± 3.5*	22.4 ± 5.6a	32.5 ± 18.6a

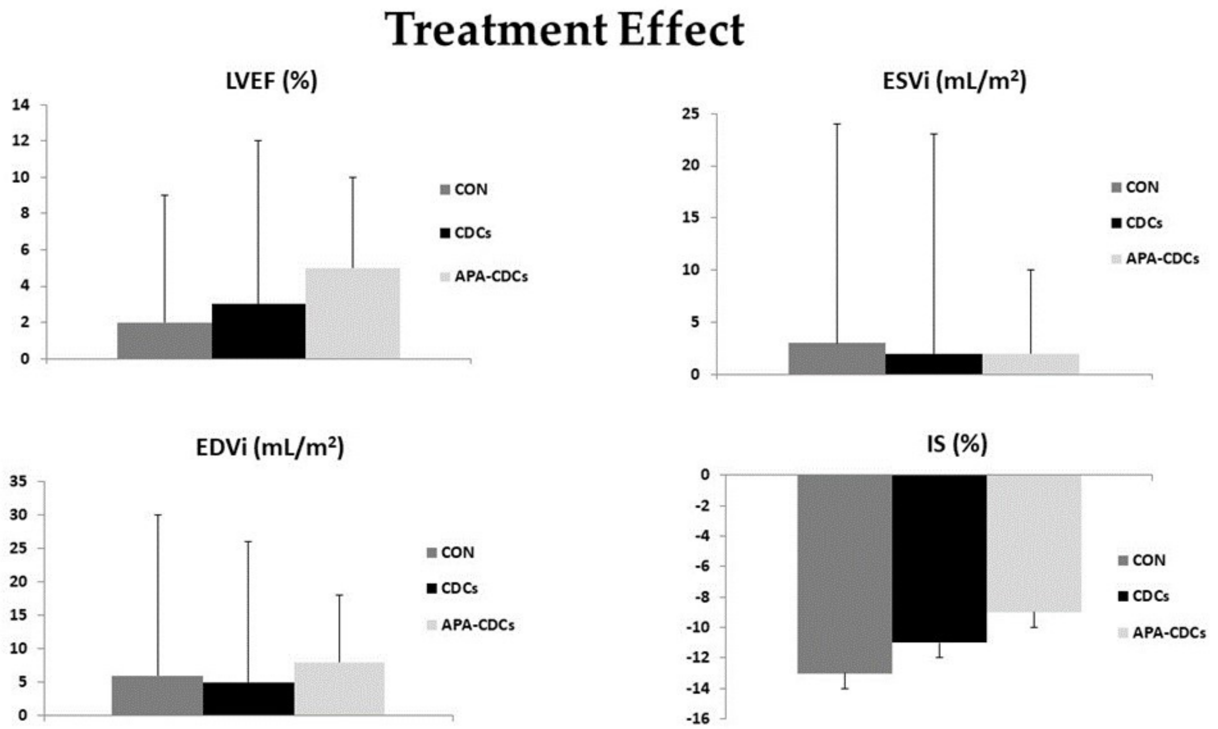

Figure S1. Treatment Effect of MRI derived cardiac function parameters: LVEF, EDVi, ESVi and IS.
